# Upper-extremity dysfunction following transradial percutaneous procedures: an overlooked and disregarded complication?

**DOI:** 10.1007/s12471-015-0749-7

**Published:** 2015-10-05

**Authors:** M.E.C.J. Hassell, J.J. Piek

**Affiliations:** Department of Cardiology, Academic Medical Center, University of Amsterdam, Amsterdam, The Netherlands

Following the introduction of transradial percutaneous coronary intervention (TR-PCI), a superficial and readily compressible access site, there have been several randomised controlled trials where TR-PCI was compared with transfemoral PCI. Currently, the radial artery is the preferred access route for catheterisation and PCI, mainly due to the lower number of access site related complications [1]. In particular in ST-segment elevation myocardial infarction patients, TR-PCI was associated with lower rates of mortality, and major and access site bleeding compared with the transfemoral approach [2−5]. Bleeding is among the most common in-hospital complications of PCI and is independently associated with increased mortality [6, 7]. Moreover, TR-PCI allows fast mobilisation of the patient and aids in reducing the in-hospital stay of STEMI patients [8].

Despite these clinical advantages, TR-PCI is technically more challenging due to the complex anatomical variability of nerves and blood vessels in the upper extremity. The radial artery is more susceptible to vasospasm, in particular in elderly, female and diabetic patients. This is often ascribed to the smaller calibre of the artery. Complications following TR-PCI include radial artery spasm, radial artery occlusion, access site bleeding, perforation, dissection, compartment syndrome, pseudo-aneurysm, arteriovenous fistula, infection and inflammation, swelling and pain. Of these complications, radial artery occlusion is the most common with a pooled incidence rate of up to 5 %. This is often asymptomatic or subclinical due to the collateral circulation of the hand. However, these described complications may ultimately result in upper extremity dysfunction in patients.

Zwaan and colleagues performed an extensive systematic review and meta-analysis on all access site complications and subsequent upper extremity dysfunction following TR-PCI and cardiac catheterisation [9]. Upper extremity dysfunction was defined as loss of strength, sensory loss, coordination and/or loss of active range of motion, ascertained by patient history and/or through physical examination. Other reported complications of TR-PCI included upper extremity ischaemia, pain, radial artery spasm, radial artery occlusion, access site bleeding, access site haematoma, perforation, dissection, swelling, compartment syndrome, pseudo-aneurysm, arteriovenous fistula, and infection/inflammation. A total of 176 studies were found eligible, including 14 articles on the incidence of upper extremity dysfunction. The authors report a pooled incidence of upper extremity dysfunction following TR-PCI and cardiac catheterisation of 0.32 % (0.10–1.01). There are some discrepancies among the included studies on how upper extremity dysfunction was assessed, which varies from investigating the handgrip strength to evaluating functional loss by history of follow-up. In particular in the former situation, this could result in underestimation of upper extremity dysfunction. Thus, it is questionable and a limitation of the study to lump these studies together. However, restricting the included studies to a more uniform assessment of upper extremity dysfunction would severely limit the eligible studies. Other frequently reported complications following TR-PCI and catheterisations included pain with a mean pooled incidence of 7.65 % (4.51–12.67), early radial artery occlusion with a pooled incidence of 3.45 (2.59–4.58) and late radial artery occlusion with a pooled incidence of 3.34 % (2.57–4.32).

Recently Van Leeuwen et al. prospectively investigated upper limb function in 338 patients undergoing coronary catheterisation, including 85 % with a radial and 15 % a femoral approach [10]. Patients had to complete two questionnaires, including a self-reported shortened version of the Disabilities of Arm, Shoulder, and Hand and Cold Intolerance Symptom Severity questionnaire, prior to catheterisation and at 30-days follow-up. In their study, upper limb function assessed by both questionnaires did not change significantly over time (prior to catheterisation to 30 days) when the operator choose a transradial approach. Moreover, the number of procedure-related extremity symptoms that persisted during 30-day follow-up was not different between the two access groups (transradial access 10.5 %, transfemoral 11.5 %; *p* = 0.82). Although these results suggest that upper limb function is not jeopardised in a transradial approach, it should be noted that the two patient groups differed significantly in sample size.

Also, it is unclear whether a questionnaire or a hand-grip strength would be a better measurement of upper extremity dysfunction. More appropriate insights into upper extremity dysfunction could be obtained by using a questionnaire in combination with an objective measurement of motor skills and sensory function measured prior to the procedure and at follow-up.

In summary, the findings of the aforementioned studies indicate the safety of TR-PCI with respect to the incidence of upper extremity dysfunction. Nevertheless, definite conclusions cannot be made based on the current literature and larger sized trials of a prospective design using uniform methods for determining upper extremity dysfunction are needed. Particularly in patients with an abnormal dual arterial supply, the incidence of upper extremity dysfunction needs to be considered as these patients have a higher risk of complications.

## Funding

None.

## Conflict of interests

None declared.
